# Dietary changes in migrant adolescents with increasing length of stay in Australia and associated risk of wheeze – a retrospective, cross sectional study

**DOI:** 10.1186/s12887-015-0420-x

**Published:** 2015-08-26

**Authors:** Lisa G. Wood, Marivic Lagleva, Smita Shah, Bronwyn S. Berthon, Sally Galbraith, Richard Henry, Helen Kepreotes, Peter G. Gibson

**Affiliations:** Centre for Asthma and Respiratory Diseases, Hunter Medical Research Institute, University of Newcastle, Newcastle, New South Wales Australia; Primary Health Care Education and Research Unit, Western Clinical School and School of Public Health, University of Sydney, Sydney, New South Wales Australia; School of Mathematics and Statistics, University of New South Wales, Sydney, New South Wales Australia; University of New South Wales, Sydney, NSW Australia; Department of Nutrition and Dietetics, Sydney Children’s Hospital Network, Randwick Campus, Sydney, New South Wales Australia; Department of Respiratory and Sleep Medicine, John Hunter Hospital, Kookaburra Circuit, New Lambton Heights, NSW 2305 Australia

**Keywords:** Paediatrics, Migration, Nutrition, Saturated fat, Fibre

## Abstract

**Background:**

Recent studies have reported that asthma prevalence increases on migration to Australia. We hypothesised that changes in dietary intake contribute to this phenomenon. The aim of this study was to assess dietary intake in relation to migration status, length of stay in Australia and the association with self-reported wheeze.

**Methods:**

Students (*n =* 144) in a multicultural high school in Western Sydney completed the asthma symptoms ISAAC video questionnaire (AVQ3.0), spirometry and allergy skin prick tests. A dietitian administered a’Food Frequency’ and ‘Food Habits’ questionnaire and a dietary history interview.

**Results:**

Students who spoke a language other than English, consumed a traditional or mixed dietary pattern, with lower consumption of saturated fat, compared to students who spoke English only. Saturated fat intake increased and fibre intake decreased with length of time in Australia. Intake of foods high in saturated or trans fatty acids were positively associated with length of stay in Australia. No associations between nutrient intake or whole food intake and self-reported wheeze were observed.

**Conclusion:**

As time progressed, dietary intake of immigrant children changed. While this was not associated with the development of wheeze in the students in this cohort, these changes are likely to have negative health consequences.

## Background

The prevalence of asthma in Australian children is one of the highest in the world, as reported by the International Study of Asthma and Allergies in Childhood (ISAAC) [1]. The National Health Survey (NHS) 2004–5 found that asthma is the most common chronic health condition in Australian children, with 20.8 % of those aged 0 to 15 years having ever been diagnosed with asthma [2]. The Longitudinal Study of Australian Children (LSAC) followed a cohort of 4–5 year olds and showed that having wheeze or asthma doubled the risk of hospitalisation or frequent general practice visits *for any cause* and of reporting fair to poor health status over the next 2 years [3]. Elucidating the causes behind the higher asthma prevalence in Australia may provide insight into how the risk of asthma in children could be modified. As asthma is increasingly being recognised as a multifactorial condition, social, environmental and genetic factors are currently under investigation for their role in the development and progression of asthma and allergies [4]. A useful avenue of exploring both social and environmental exposures in relation to asthma risk is through the study of international immigrants [5]. Upon arrival migrant populations show different patterns of disease prevalence in comparison to the population of their new country of residence, though these differences usually disappear over time [6]. Migration studies show that when adolescents from countries with lower asthma prevalence rates migrate to Australia, their prevalence of asthma symptoms is significantly higher than their country of origin [7]. A Melbourne study of 51 high schools (*n =* 9778) found that migrant students that had stayed in Australia for 5–9 years and 10–14 years compared to those that had stayed 0–4 years had an increased chance of reporting wheeze [8]. Another Melbourne study found that the prevalence of asthma in Asian immigrants (*n =* 636) increased significantly with the length of stay in Australia [9]. We have previously reported that the prevalence of wheeze at rest was lower amongst adolescents who were recent migrants to Australia compared to those who had lived in Australia since birth [7].

Understanding the changes in environmental exposures of recent migrants may increase our understanding of the mechanisms behind the increased risk of asthma symptoms associated with migration.

Dietary patterns and nutrient intake are now recognised as a significant environmental exposure that may impact on asthma risk [10]. A number of recent reviews have examined the hypothesis that dietary changes, including decreasing antioxidant intake, decreasing n-3 polyunsaturated fat intake and increasing consumption of saturated and trans fatty acids are linked to increasing asthma prevalence [11–13]. The ISAAC phase 2 cross-sectional study consisting of 20 countries and over 50 000 school children aged 8–12 years, recently reported that fish consumption in affluent countries was associated with decreased wheeze, a common symptom of asthma. Fruit and cooked vegetables consumption in non-affluent countries was also associated with decreased wheeze. Furthermore, burger consumption of greater than 3 times per week compared to never or occasionally was associated with an increased risk of ever having asthma. In contrast, raw green vegetable intake greater than 3 times per week compared to never or occasionally was associated with a decreased risk in ever having asthma [14]. The ISAAC phase 3 study analysed data from over 300 000 adolescents (13–14 years old) from 107 centres from 51 countries and over 180 000 children (6–7 years old) from 64 centres from 31 countries. They consistently found for both age groups that an increased risk of severe asthma, rhino conjunctivitis and eczema is associated with greater than 3 times per week consumption of fast food. In contrast, greater than 3 times per week consumption of fruit is associated with a decreased risk of severe asthma, rhino conjunctivitis and eczema [15].

We hypothesised that changes in dietary intake occur in adolescents following arrival in Australia, which may be related to the development of asthma symptoms in some individuals. The aim of this study was to perform a retrospective analysis of cross-sectional data to investigate differences in dietary intake in immigrant children compared to non-immigrants, whether dietary intake changes with length of stay in Australia and whether these changes in dietary intake are related to self-reported wheeze with increasing length of stay in Australia.

## Methods

### Study design

We conducted a retrospective, cross-sectional nested study of a larger study of asthma symptoms and airway inflammation previously described [7, 16]. Adolescent subjects, aged 12–18 years, were recruited from a multicultural high school in Western Sydney, Australia, in 1997 and 1998. The high school was chosen as it has one of the highest rates of recent migrant and refugee student enrolments in Sydney, NSW, Australia. The school has an Intensive English Centre to assist students from non-English speaking backgrounds to learn English. All students in the high school were invited to participate, and all students completed the ISAAC video questionnaire. The participation rate in the original study was 78 %. 271 students were approached, and 211 completed full asthma assessment. Of the 262 students who completed the ISAAC questionnaire, 144 students completed the dietary component.

This study includes the subset of subjects (*n =* 144, 55 % of all the subjects in the original study) for whom dietary records were available. Asthma symptoms including self-reported wheeze were recorded using the validated ISAAC video questionnaire, version 3.0 (AVQ3.0) [17] which consists of five video sequences of young people with different asthma symptoms. The first three sequences show various scenes of wheezing, while the final two sequences display other asthma symptoms. The scenes depicted: 1) moderate wheezing at rest (a Caucasian girl); 2) wheezing after exercise (a Maori boy); 3) waking at night with wheezing (a Caucasian girl); 4) waking at night with coughing (an Asian boy); and 5) a severe attack of asthma with wheezing and breathlessness at rest (an Indian woman). After each video sequence, students recorded on a one-page printed answer sheet whether their breathing had ever been like that shown in the video and, if so, the frequency of such symptoms (past month, past year, ever). The video questionnaire took about 7 min to administer, and the term “asthma” was not mentioned during this time. The questionnaire was presented to students by an ethnic healthcare worker who was based at the school. The child was asked to complete the questionnaire and to ask for clarification if required. When assistance was requested by the student, this was provided in both English and in the native language of the student.

Students completed an asthma medication questionnaire, and underwent spirometry and allergy skin prick tests. Forced expiratory volume in one second (FEV_1_) and forced vital capacity (FVC) were performed by wearing nose clips in the seated position using a dry wedge spirometer (Vitalograph, Buckingham, UK), and the best of three readings was recorded.

Skin prick tests were performed on the volar aspect of the forearm, using cat fur, grass mix, Alternaria tenuis, whole egg, cow’s milk, Dermatophagoides farinae, Dermatophagoides pteronyssinus and cockroach (Bayer, Australia Ltd.). Positive (histamine 1 %) and negative (50 % glycerine) controls were used. A positive reaction was defined as a 3-mm or greater wheal diameter 15 min after skin prick. Permission to conduct this research was obtained from the Department of Schools Education and the Hunter Area Health Service and University of Newcastle Research Ethics Committees. Written informed consent was obtained from parents and students.

### Dietary assessment tools

The data collection period was from October to December 1998. A 50 min interviewer administered questionnaire was conducted by a dietitian. Interpreters were used for 38 (25 %) of the subjects interviewed. The nationality, language spoken other than English and the number of years lived in Australia, were recorded.

The diet history method [18] was used to record the usual weekly meal and snack consumption. Food intake was quantified using metric cup and spoon measures, a ruler to indicate dimensions of certain foods, photographs of commercial foods, typical household measures such as plates and bowls as well as three dimensional plastic food models. The food frequency qualitative questionnaire was intended to establish food consumption habits and was based on that used in the 1995 National Nutrition Survey [19]. Respondents were asked how often they consumed 107 food and drink items and 11 vitamin and mineral supplements over the past 12 months by estimating frequency of consumption. In addition there were 13 questions on dietary habits relating to fruit, vegetable, salt, fish and reduced fat dairy consumption. Nutrient intake was calculated using the software program DIET/1(version 4.00, 1997; Xyris Software, Brisbane, Queensland, Australia) which used the Australian NUTTAB 95 food composition database [20].

Take away food and snack food practices were also noted. The supervisor of the school canteen was contacted to obtain lists of menus, recipes and to inspect available food products. Dietary patterns were assessed by the dietitian as traditional, mixed and westernised. The definition for a traditional diet was high intake of fruits, vegetables and wholegrains with low intakes of red meat. The definition of a mixed dietary pattern was that the student ate traditional food at home but westernised food at school and frequently consumed takeaway foods. A westernised diet was defined as high in processed foods such as refined sugars, refined vegetable oils and fatty meats.

### Statistical analysis

Group comparisons were made using Mann–Whitney tests for continuous variables and Pearson’s chi-squared test for categorical variables. Multiple linear regression was used to do analyses of English language grouping association with nutrient or whole food intake adjusted for sex and age. Multiple linear regression was applied to explore associations between the intake of whole foods or nutrients and years living in Australia, while adjusting for control variables age and sex. Multiple logistic regression was applied to explore associations between vegetable intake and years living in Australia and between wheeze and dietary intake, while adjusting for years lived in Australia, age and sex. All exploratory analyses were carried out with the statistical program software R version 2.12.1.

## Results

### Subject characteristics

Data were analysed from a total of 144 high school students from whom both asthma symptoms and dietary data were available. Subject characteristics are summarised in Table 1. 24.3 % of subjects had wheeze at rest. The median interquartile range (IQR) time of residence in Australia was 3 (0.7–12.3) years and 65 % spoke another language other than English. The students came from 30 different countries that were collapsed into regions. The nationalities represented in the school were Middle Eastern 57/144 (40 %), Australian 40/144 (28 %), Asian 26/144 (18 %) and other mixed nationalities 21/144 (15 %).

**Table 1:** Subject characteristics

N	144
Age^a^	13.6 (12.8–15.2)
Sex (%Males)	53.5
BMI (kg/m^2^)	20.2 (18.1–22.8)
FEV_1_, L	2.85 (2.40–3.40)
FVC, L (*n =* 116)	3.18 (2.79–3.80)
FEV_1_/FVC (*n =* 116)	0.90 (0.85–0.95)
DRS (%fall/mL)	0.22 (0.07–0.44)
Asthma Symptoms (%Yes)	28.5
Atopy (%Yes)	51.4
Wheeze (%Yes)	24.3
Asthma medication usage (%Yes)^b^	12.5
Years in Australia	3.0 (0.7–12.3)
Language (%English only)	34.7

*BMI* Body mass index, *FEV1* forced expiratory volume in 1 s; *FVC* forced vital capacity; *DRS* dose response slope

^a^All data are presented as Median (IQR) unless otherwise stated. ^b^Asthma medications include: Alupent, Asmol, Atrovent, Becloforte, Becotide, Bricanyl, Flixotide, Pulmicorte, Respolin, Serevent, Theophylline, Ventolin

Table 2 describes the results of a regression analysis examining the association between nutrient intake and immigrant status classified according to language spoken at home. Both carotene and retinol equivalent intake was higher for the English only speaking students. Vitamin C intake tended to be higher for those that spoke a language other than English and this was borderline significant. Fibre intake tended to be lower in the English only speaking students and this was also borderline statistically significant.

**Table 2:** Regression analysis examining the association between nutrient intake (as outcome) and immigrant status classified according to language spoken at home (language other than English *n =* 94, or English only *n =* 56) (as predictor), adjusted for age and sex

Nutrient intake	Unadjusted β-coefficient (*n =* 144)	95 % CI	*P*	β-coefficient adjusted for age and sex (*n =* 138)	95 % CI	*P*
Antioxidants						
Carotene (ug)	982.3	371.8 – 1592.7	0.002	1109.5	433.6 – 1785.9	0.001
Retinol equivalents (ug)	173.3	18.1 – 328.5	0.029	182.9	11.5 – 354.3	0.037
Vitamin C (mg)	−31.4	−68.7 – 5.9	0.099	−40.1	−80.3 – 0.1	0.051
Dietary fat						
Total fat (g)	−9.3	−23.0 – 4.5	0.186	−13.1	−28.1 – 1.9	0.088
%Saturated fat^a^	1.6	0.5 – 2.7	0.006	1.1	−0.1 – 2.3	0.072
%Monounsaturated fat^a^	0.3	−0.6 – 1.1	0.552	0.3	−0.6 – 1.2	0.545
%Polyunsaturated fat^a^	0.3	−0.04 – 0.7	0.084	0.3	−0.1 – 0.7	0.153
Other						
Fibre (g)	−3.6		0.022	−3.3		0.055

^a^Expressed as a percentage of total energy intake

Tables 3 and 4 describe the results of a regression analysis examining the association between whole dietary intake and immigrant status classified according to language spoken at home. Some whole foods high in saturated fat such as crisps and cake were consumed at higher levels in those that spoke English only. Of those that spoke a language other than English, more students consumed a traditional or mixed dietary pattern, while in the only English speaking group, more students consumed a westernised dietary pattern.

**Table 3:** Regression analysis examining the association between whole food intake (as outcome) and immigrant status classified according to language spoken at home (Language other than English or English only) (as predictor), adjusted for age and sex

Whole food intake (serves/day)	Unadjusted β-coefficient (*n =* 144)	95 % CI	*P*	β-coefficient adjusted for age and sex (*n =* 144)	95 % CI	*P*
Foods high in Antioxidants						
Fruit	−0.1	−0.3 – 0.2	0.477	−0.1	−0.3 – 0.2	0.558
Nuts	0.02	−0.1 – 0.1	0.585	0.01	−0.1 – 0.1	0.920
Tea	−0.2	−0.3 – 0.01	0.060	−0.1	−0.3 – 0.1	0.297
Foods high in Saturated/Trans fats						
Takeaway	−0.01	−0.1 – 0.02	0.430	−0.03	−0.1 – 0.01	0.174
Pies	0.1	−0.01 – 0.1	0.111	0.03	−0.1 – 0.1	0.451
Chocolate	0.1	−0.1 – 0.2	0.404	0.1	−0.1 – 0.2	0.353
Cake	0.2	0.1 – 0.4	0.009	0.2	−0.01 – 0.3	0.058
Ice cream	0.1	−0.05 – 0.2	0.258	0.03	−0.1 – 0.2	0.670
Crisps	0.2	0.05 – 0.3	0.009	0.2	0.03 – 0.3	0.021
Foods high in omega-3 fats						
Fish	−0.01	−0.1 – 0.1	0.872	−0.027	−0.1 – 0.04	0.421

**Table 4:** Frequencies between dietary intake and immigrant status classified according to language spoken at home (Language other than English or English only) (*n =* 138)

Vegetable intake	Language other than English (*n =* 88)	English only (*n =* 50)	*P*
0.5 serves/day	67 (76)	34 (48)	0.403
>0.5 serves/day	21 (24)	16 (32)	
Dietary Pattern			
Traditional	27 (31)	2 (4)	<0.001
Mixed	58 (66)	7 (14)	
Westernised	3 (3)	41 (82)	

Data presented as n(%)

**Fig. 1:**
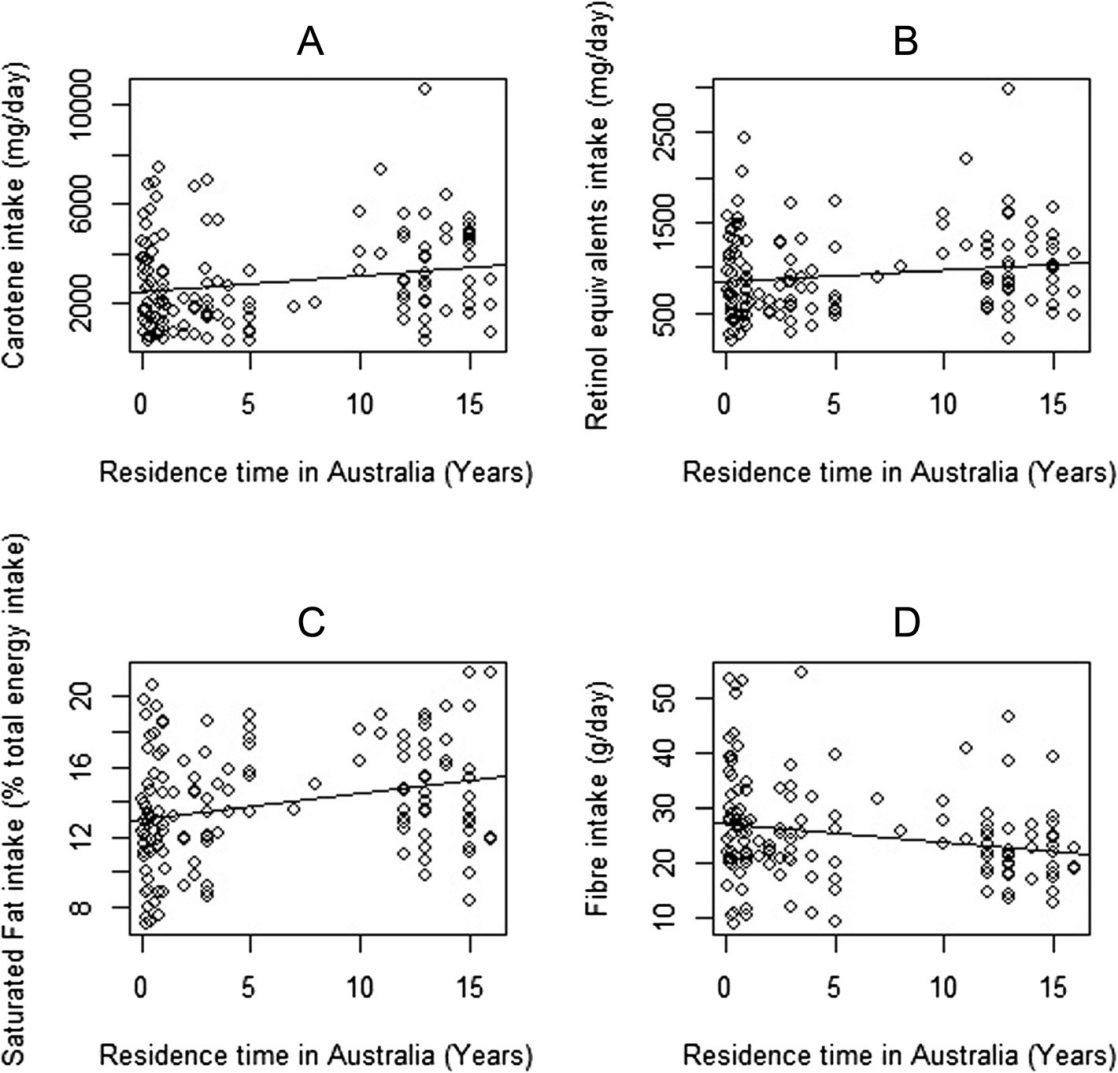
Correlation between length of time in Australia and nutrient intake, including **a** Carotene (coefficient = 67.630 p-value = 0.01), **b** Retinol equivalents (coefficient = 12.531 p-value = 0.058), **c** %Saturated Fat (coefficient = 0.146 p-value = 0.002) and **d** Fibre (coefficient = 0.324 p-value = 0.013)

**Fig. 2:**
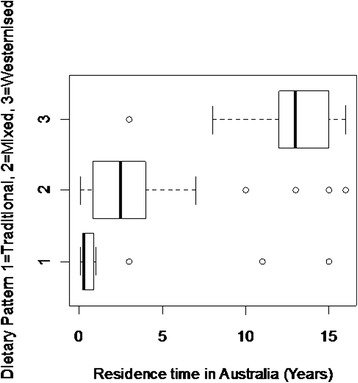
Association between dietary pattern and length of time in Australia

Table 5 describes the association between nutrient intake and time residing in Australia. Carotene and retinol equivalents showed a positive association with time in Australia that persisted after adjusting for age and sex [Fig. 1a and b]. No association was found with vitamin C. Saturated fat intake increased as length of time in Australia increased and this association remained after controlling for age and sex [Fig. 1c]. Conversely, fibre intake decreased with increasing length of stay in Australia in both the unadjusted and adjusted models [Fig. 1d]. In the analysis of whole foods (Tables 6 and 7), foods high in saturated or trans fatty acids were positively associated with time in Australia, including pies, cakes and crisps. After adjustment for age and sex, pies and crisps remained significantly positively associated with time in Australia. Figure 2 demonstrates that dietary pattern was associated with time in Australia. No associations between nutrient intake or whole food intake and self-reported wheeze were observed (Tables 8, 9 and 10).

**Table 5:** Regression analysis examining the association between nutrient intake (outcome) and length of time in Australia (predictor)

Nutrient intake	Unadjusted β-coefficient (*n =* 144)	95 % CI	*P*	β-coefficient adjusted for age and sex (*n =* 144)	95 % CI	*P*
Antioxidants						
Carotene (ug)	67.6	16.0 – 119.2	0.011	77.8	19.5 – 136.0	0.009
Retinol equivalents (ug)	12.5	−0.4 – 25.5	0.058	15.4	0.9 – 29.9	0.037
Vitamin C (mg)	0.2	−2.9 – 3.2	0.909	−0.6	−4.03 – 2.7	0.706
Dietary fat						
Total fat (g)	−0.6	−1.7 – 0.6	0.318	−0.6	−1.9 – 0.6	0.325
%Saturated fat^a^	0.1	0.1 – 0.2	0.002	0.1	0.02 – 0.2	0.021
%Monounsaturated fat^a^	0.01	−0.1 – 0.1	0.733	0.01	−0.1 – 0.1	0.752
%Polyunsaturated fat^a^	0.01	−0.02 – 0.03	0.664	0.003	−0.03 – 0.04	0.874
Other						
Fibre (g)	−0.3	−0.6 – −0.1	0.013	−0.3	−0.6 – −0.1	0.021

^a^Expressed as a percentage of total energy intake

**Table 6:** Regression analysis examining the association between whole food intake (outcome) and length of time in Australia (predictor)

Whole food intake (serves/day)	Unadjusted β-coefficient (*n =* 144)	95 % CI	*P*	β-coefficient adjusted for age and sex (*n =* 144)	95 % CI	*P*
Foods high in Antioxidants						
Fruit	0.01	−0.01 – 0.03	0.269	0.01	−0.01 – 0.03	0.318
Nuts	0.002	−0.01 – 0.01	0.602	−0.001	−0.01 – 0.01	0.842
Tea	−0.02	−0.03 – -0.01	0.008	−0.01	−0.03 – 0.003	0.131
Foods high in Saturated/Trans fats						
Takeaway	0.001	−0.003 – 0.004	0.687	−0.0001	−0.004 – 0.003	0.916
Pies	0.01	0.002 – 0.02	0.009	0.01	0.001 – 0.015	0.041
Chocolate	0.01	−0.003 – 0.02	0.146	0.01	−0.01 – 0.02	0.339
Cake	0.02	0.004 – 0.03	0.010	0.01	−0.003 – 0.02	0.125
Ice cream	0.01	−0.001 – 0.02	0.099	0.01	−0.01 – 0.02	0.377
Crisps	0.02	0.01 – 0.03	<0.001	0.02	0.01 – 0.03	0.003
Foods high in omega-3 fats						
Fish	0.003	−0.001 – 0.01	0.158	0.002	−0.004 – 0.01	0.511

**Table 7:** Frequencies of dietary intake and categories of length of time in Australia (*N =* 144)

	Time in Australia			
Vegetable intake	<= 2 years (*n =* 61)	>2 years, <Lifetime (*n =* 38)	Lifetime (*n =* 45)	*P*
0.5 serves/day	50 (82)	24 (63)	41 (91)	0.094
>0.5 serves/day	11 (18)	14 (37)	4 (9)	
Dietary Pattern				
Traditional	28 (46)	3 (8)	1 (2)	
Mixed	33 (54)	31 (82)	4 (9)	NA^a^
Westernised	0	4 (10)	40 (89)	

^a^Not Available as a p-value cannot be obtained with the chi-squared test for a cell having zero value

**Table 8:** Logistic regression analysis examining the association between wheeze (outcome) and nutrient intake (predictor)

Nutrient intake	Unadjusted odds ratio (*n =* 144)	95 % CI	*P*	Adjusted^b^ odds ratio (*n =* 144)	95 % CI	*P*
Antioxidants						
Carotene (ug)	1.0	1.0 – 1.0	0.930	1.0	1.0 – 1.0	0.555
Retinol equivalents (ug)	1.0	1.0 – 1.0	0.829	1.0	1.0 – 1.0	0.743
Vitamin C (mg)	1.0	1.0 – 1.0	0.090	1.0	1.0 – 1.0	0.063
Dietary fat						
Total fat (g)	1.0	1.0 – 1.0	0.347	1.01	1.0 – 1.0	0.217
%Saturated fat^a^	1.1	1.0 – 1.2	0.244	1.04	0.9 – 1.2	0.593
%Monounsaturated fat^a^	1.1	1.0 – 1.3	0.117	1.2	1.0 – 1.4	0.111
%Polyunsaturated fat^a^	0.9	0.6 – 1.3	0.623	0.9	0.6 – 1.3	0.535
Other						
Fibre (g)	1.0	0.9 – 1.0	0.468	1.0	1.0 – 1.0	0.853

^a^Expressed as a percentage of total energy intake, ^b^Adjusted for age, sex and length of time in Australia

**Table 9:** Logistic regression analysis examining the association between wheeze (outcome) and whole food intake (predictor)

Whole food intake	Unadjusted odds ratio (*n =* 144)	95 % CI	*P*	Adjusted^a^ odds ratio (*n =* 144)	95 % CI	*P*
Foods high in Antioxidants						
Fruit	0.9	0.5–1.6	0.635	0.9	0.5–1.6	0.645
Nuts	0.9	0.2–3.4	0.680	0.8	0.2–3.5	0.783
Tea	1.0	0.4–2.2	0.863	1.2	0.5–2.7	0.705
Foods high in Saturated/Trans fats						
Takeaway	0.2	0.01–6.2	0.129	0.1	0.002–6.1	0.286
Pies	0.9	0.2–4.8	0.518	0.5	0.1–3.4	0.479
Choc	0.9	0.3–2.7	0.597	0.8	0.2–2.6	0.677
Cake	1.4	0.6–3.2	0.723	1.1	0.5–2.8	0.777
Ice cream	0.8	0.2–2.6	0.559	0.6	0.2–2.2	0.490
Crisps	1.5	0.6–3.8	0.583	1.1	0.4–3.0	0.823
Foods high in omega-3 fats						
Fish	1.3	0.1–11.9	0.843	0.980	0.1–11.2	0.987

^a^Adjusted for age, sex and length of time in Australia

**Table 10:** Frequencies of wheeze at rest and dietary intake (*N =* 144)

Vegetable intake	Wheeze at rest = No (*n =* 109)	Wheeze at rest = Yes (*n =* 35)	*P*
0.5 serves/day	81 (74)	24 (69)	0.665
>0.5 serves/day	28 (26)	11 (31)	
Dietary Pattern			
Traditional	26 (24)	6 (17)	0.355
Mixed	53 (49)	15 (43)	
Westernised	30 (27)	14 (40)	

## Discussion

In our cross-sectional analysis we have described the differences in dietary intake in Australian households which speak a language other than English, compared to English-only speaking households. We have demonstrated that those households who spoke a language other than English, consumed a traditional or mixed dietary pattern, with lower intake of foods containing saturated fat. We have also shown that dietary changes occur over time when adolescents migrate from a developing country to a western country, with increased consumption of saturated fat and decreased intake of fibre. This was driven by an increase in fast-foods including pies and crisps. Contrary to our hypothesis, no associations between nutrient intake or whole food intake and self-reported wheeze were observed.

The consequence of the changes in dietary pattern that we observed in newly arrived migrant students is the potential exposure to several pro-inflammatory nutrients. Dietary fat, in particular saturated fatty acids, can activate an innate immune response, involving activation of Toll-like receptors, which are cell surface receptors that are traditionally known to be activated by bacteria [13]. As a result, an inflammatory cascade is initiated, involving activation of the transcription factor NFκB and increased expression of pro-inflammatory cytokines such as IL-6 and TNFα. Reduced intakes of fibre can also promote inflammation. A proposed mechanism involves butyrate, a short chain fatty acid (SCFA), produced as microbiota in the gut digest dietary fibre. This activates the peroxisome proliferator-activated receptor-α (PPARα) which then inhibits NFкB activity [21]. SCFAs also interact with receptors from a family of G protein-coupled receptors [22, 23]. G protein-coupled receptor 43 (GPR43) activation by acetate, another SCFA produced by microbiota fermentation of fibre, caused apoptosis in neutrophils and reduced inflammatory responses in mice models of airway inflammation, further supporting the anti-inflammatory role of fibre [23].

This inflammatory environment may contribute to the development of various inflammatory diseases. Indeed, we hypothesised that the increased prevalence of wheeze on migration to a western country that we have previously reported in this cohort [7], may have been associated with these dietary changes. Intervention studies have shown that nutritional modulation affects asthma outcomes. For example, a high antioxidant diet reduced exacerbation risk and improved lung function compared to a low antioxidant diet [24]. Conversely, a high-fat meal can worsen airway inflammation in asthma [25]. A number of population-based studies have also shown associations between western diets (including reduced fruit and vegetable intake, increased intake of fat, processed foods and fast foods) and increased asthma risk in children [26–28]. Furthermore, children who reported eating fruit more than once a day had higher lung function than children who reported never eating fruit [29]. We have also reported an association between high fat and low fibre diets and worse airway inflammation and lung function in asthma [30]. It is important to acknowledge that while this data was collected some time ago, migration into Australia from developing countries continues to occur, thus people continue to be exposed to a different environment (including dietary intake) from their country of origin. We believe that these results are still relevant and are able to inform us that diet does change on migration and our results are able to explain the nature of the dietary change.

The observational study design employed in this investigation is limited to detecting associations, and as it was cross-sectional is only able to show a snapshot of dietary intake and asthma symptoms at one point in time. The data for this study was collected as part of a previously conducted larger study. Not all students in the original study completed the dietary assessment reported in this study, thus a full data set was available for 55 % of the original study population. This could introduce bias and limit the generalisability of the results, though the extent of this is unknown. While our analysis didn’t identify an association between self-reported wheeze and dietary changes in immigrant children, it is possible that the study was underpowered to detect such an association. The selection of this small sample is also subject to bias as it was limited to convenience sample of subjects who had provided both asthma symptoms and dietary data. Furthermore there is a potential for selection bias in that the dietary intakes of these subjects may not be completely representative of their population groups. Further investigation of this phenomenon in a larger cohort, also looking at trends in dietary intake over time is warranted. This study displays many strengths including the use of a diverse range of validated assessment tools, rigorous statistical analysis and access to a unique population.

## Conclusions

In summary, this study has shown that dietary patterns and nutrient intake of adolescent migrants from a developing country differ to the host population. We have also elucidated the nature of the dietary change and concluded that these changes are likely to promote an inflammatory environment. While we didn’t identify any association between dietary change and increased wheeze, it is likely that there will be negative health consequences arising from this type of change in food consumption, which should be monitored in other settings. Our study provides useful insight into the changes that occur upon migration and suggest that investigation of health and disease in migration may shed some light into the environmental risks associated with asthma and reasons behind discordant international asthma prevalence rates.

## Abbreviations

BMI: Body mass index
DRS: Dose response slope
FEV1: Forced expiratory volume in 1 s
FVC: Forced vital capacity
GPR43: G protein-coupled receptor 43
IQR: Interquartile range
ISAAC: International Study of Asthma and Allergies in Childhood
LSAC: The Longitudinal Study of Australian Children
NHS: The National Health Survey (NHS)
PPARα: Peroxisome proliferator-activated receptor-α
SCFA: Short chain fatty acid
